# Understanding the Increase in Life Expectancy in Hong Kong: Contributions of Changes in Age- and Cause-Specific Mortality

**DOI:** 10.3390/ijerph16111959

**Published:** 2019-06-02

**Authors:** Yan Zheng, Qingsong Chang, Paul Siu Fai Yip

**Affiliations:** Department of Social Work and Social Administration, The University of Hong Kong, Hong Kong, China; u3005433@hku.hk (Y.Z.); changqs@hku.hk (Q.C.)

**Keywords:** mortality, life expectancy, public health policy

## Abstract

To assess the mechanism responsible for the improvement in life expectancy in Hong Kong over the past several decades, Arriaga’s decomposition method was applied to quantify the influence of the age structure and the leading causes of death on the increase in life expectancy in Hong Kong from 1986 to 2015. Our results showed that, during the observed period, the major contribution to the improvement in life expectancy in Hong Kong was attributable to the older population for both males and females. Contributions of malignant neoplasms in the period of 1986–1995, 1996–2005 and 2006–2015 were 0.613, 0.279 and 0.887 years in males and 0.391, 0.312 and 0.226 years in females, respectively. For circulatory diseases, the corresponding figures were 1.452, 0.202 and 0.832 years in males and 0.675, 0.192 and 1.069 years in females, with the largest contribution also shifting to older ages. However, these positive contributions were partly offset by the negative contribution of pneumonia, especially among those at advanced ages. Furthermore, although the impact was limited, attention should also be paid to the prevalence of some chronic diseases among the younger age groups in recent years.

## 1. Introduction

Over the last several decades, life expectancy has been rising substantially throughout the world. Life expectancy is an important summary measure of mortality. It reflects the health conditions of all age groups and covers multiple health dimensions [1]. Also, it is often an early predictor of societal issues [2]. Therefore, research on life expectancy is of crucial importance for exploring the health status of communities and for strengthening social and health policy decisions.

It is well known that the increase in life expectancy is the result of a decline in mortality. However, the relative contributions of mortality rates to the change in life expectancy are not necessarily the same across all age groups, and that is the main reason why many previous studies have tended to explore the growth mechanism of life expectancy through age- and cause-specific decomposition [3,4,5,6,7]. These studies reveal the substantial importance of understanding which age groups and leading causes of death are responsible for the change in life expectancy, as such an understanding could lead to more targeted public health policies being developed to promote better population health.

**Figure A1:**
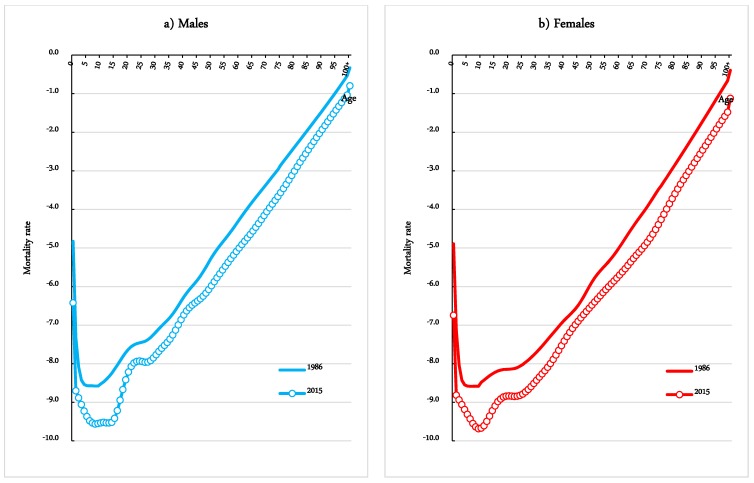
Mortality change in Hong Kong between 1986 and 2015: (**a**) Males and (**b**) Females.

In Hong Kong, with the reduction in mortality rates (Appendix A; Figure A1), life expectancy has also experienced a remarkable increase. Over the past three decades, life expectancy in Hong Kong has increased from 74.1 years in 1986 to 81.4 years in 2015 in males, and from 79.4 years to 87.3 years in females [8]. In recent decades, several studies have been conducted to explore the life expectancy of the Hong Kong population [9,10,11,12]. These studies described the change in life expectancy of patients who suffered from certain diseases—for example, rheumatic diseases—or discussed the healthy life expectancy in Hong Kong, which provided a meaningful understanding of Hong Kong residents’ health status. However, the mechanism of the improvement in life expectancy in Hong Kong has thus far not been examined.

To reveal the growth mechanism of life expectancy in Hong Kong over the past decades, contributions of mortality change by age group and selected causes of death to the change in life expectancy have been assessed. The paper is structured as follows: A brief description of the data source and the decomposition method is provided in the Materials and Methods section; contributions of the age structure and the leading causes of death to the increase in life expectancy in Hong Kong from 1986 to 2015 are displayed in the Results section; and the Conclusion and potential future directions, according to the present work, are given in the final section. Findings in the study will provide useful information in allocating health resources more effectively and implementing health-related public policies more directly, yielding multi-practical implications.

## 2. Materials and Methods

Mortality data for the major causes of death for the period under study (1986 to 2015) were obtained from the registered death dataset through the Census and Statistics Department of Hong Kong (HKCSD). Due to a mandatory reporting system, death registration in Hong Kong is almost intact [9]. Therefore, it could be assumed that the vital data used in this study were reliable. Since the data were publicly available, this study was exempt from ethics approval.

The International Classification of Diseases (ICD) categorizes the causes of death into subgroups. Three ICD revisions were included in the study period (1986–2000 ICD 9, 2001–2009 ICD 10 and 2010 onwards ICD update) (Appendix B; Table A1). To improve the comparability over time, standardized mortality rates were calculated based on the population of Hong Kong in 2001.

With the use of annual mortality rates, life expectancy was calculated with the construction of complete life tables. Improvement in life expectancy is the result of a decline in mortality rates. However, in view of the change in mortality over different age groups or disease categories, it is important to examine the change in life expectancy in more detail.

In this study, the method developed by Arriaga was applied in order to conduct the decomposition analyses [13,14]. Arriaga’s decomposition method is effective in evaluating the contributions of mortality change in each age and cause to the overall change in life expectancy over a given period. For calculation, the following formula can be used:

(1) $${}_{n}\Delta_{x}=\frac{l_{x}^{1}}{l_{0}^{1}}\times \left(\frac{{}_{n}L_{x}^{2}}{l_{x}^{2}}-\frac{{}_{n}L_{x}^{1}}{l_{x}^{1}}\right)+\frac{T_{x+n}^{2}}{l_{0}^{1}}\times \left(\frac{l_{x}^{1}}{l_{x}^{2}}-\frac{l_{x+n}^{1}}{l_{x+n}^{2}}\right)$$

For the open-aged group, the contribution can be expressed as:

(2) $${}_{\infty }\Delta_{x}=\frac{l_{x}^{1}}{l_{0}^{1}}\times \left(\frac{T_{x}^{2}}{l_{x}^{2}}-\frac{T_{x}^{1}}{l_{x}^{1}}\right)$$

where _*n*_Δ_*x*_ is the age-specific difference between life expectancies at time 1 and time 2. $l_{x}^{1}$, ${}_{n}L_{x}^{1}$ and $T_{x}^{1}$ denote the number of survivors at age *x*, the number of person-years lived between age *x* and *x* + *n*, and the total number of person-years lived after age *x*, respectively. These variables can be obtained from the life tables at time 1, while $l_{x}^{2}$, ${}_{n}L_{x}^{2}$ and $T_{x}^{2}$ are the variables in the life tables corresponding to time 2.

The disease-specific contribution between time 1 and time 2 can be calculated by the following formula:

(3) $${}_{n}\Delta_{x}^{i}={}_{n}\Delta_{x}\times \frac{{}_{n}m_{x}^{i}\left(2\right)-{}_{n}m_{x}^{i}\left(1\right)}{{}_{n}m_{x}\left(2\right)-{}_{n}m_{x}\left(1\right)}$$

In the formula, ${}_{n}m_{x}^{i}\left(1\right)$ and ${}_{n}m_{x}^{i}\left(2\right)$ denote the mortality of specific diseases at age *x* at time 1 and time 2. ${}_{n}m_{x}\left(1\right)$ and ${}_{n}m_{x}\left(2\right)$ represent the all-cause mortality at age *x* at time 1 and time 2, respectively. ${}_{n}\Delta_{x}$ denotes the age-specific difference of life expectancies between time 1 and time 2. With the application of Arriaga’s method, the total change in life expectancy can be decomposed into age and cause-specific components.

## 3. Results

Over the observation period, for both males and females, mortality rates experienced significant reduction across all ages, as shown in Table A1, which resulted in a remarkable increase in the life expectancy of residents in Hong Kong. In this section, we examine the growth mechanism of the Hong Kong life expectancy, and analyze contributions of mortality change by age and selected causes of death to the change in life expectancy in Hong Kong from 1986 to 2015.

Mortality rates for selected causes of death from 1986 to 2015 in Hong Kong are provided in Table 1 for both males and females. It can be observed that for both genders, most of the deaths were due to diseases of malignant neoplasms and the circulatory system, including cerebrovascular diseases and heart diseases. Death from respiratory diseases also accounted for an important but lower percent of all deaths for both males and females. In addition, cause-specific mortality rates were different across the two genders. In the case of malignant neoplasms, males generally had a higher mortality rate from malignant neoplasms than females. The mortality from malignant neoplasms in males increased from 172.4 in 1986 to 247.4 in 2015, higher than that in females (117.1 in 1986 and 151.5 in 2015). The number of deaths that resulted from other diseases—for instance, infectious diseases—was relatively small.

### 3.1. Contributions of Mortality Change by Age Group

**Figure 1:**
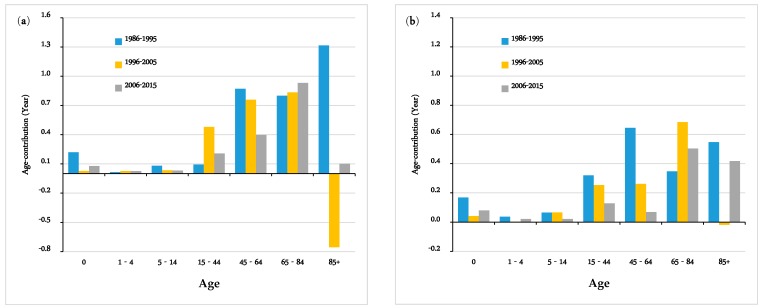
Arriaga’s decomposition of the change in life expectancy by age group between 1986 and 2015 in Hong Kong: (**a**) Males; (**b**) Females.

Figure 1 reflects the contributions of mortality change to the improvement in life expectancy in Hong Kong by each age group over the observed period between 1986 and 2015. For both males and females, life expectancy increased over the past three decades (3.39, 1.40 and 1.77 years in males and 2.13, 1.29 and 1.24 years in females for 1986–1995, 1996–2005 and 2006–2015, respectively), although the rate of improvement in life expectancy over the latter two decades (1996–2005 and 2006–2015) slowed.

During 1986–1995, the observed increase in female life expectancy in Hong Kong was largely attributed to the decline in mortality among those aged 45–64 years, accounting for 30.3% of the total improvement in female life expectancy. However, in 2006–2015, the contribution rate of this age group dropped significantly to 5.6%. On the contrary, in the latter two decades of the study (1996–2005 and 2006–2015), the older females aged 65–84 years made substantial positive contributions (53% and 40.5% to the total increase in female life expectancy, respectively). In addition, a further decline in the mortality of older females aged 85 and above aged also had an important role in the increase in life expectancy in Hong Kong over the observed period, with the contribution percentage increasing from 25.7% to 33.6%. In fact, the shift in the contribution pattern was mainly driven by the marked improvement of health status among older females in Hong Kong.

The male contribution pattern experienced a generally similar change over the past decades, with the peak shifting to the older group aged 65–84, except in the case of the first decade when contributions of old males at advanced ages was greater than that of the adult groups. Besides, compared with females, the positive impact of males aged 45–64 was much larger, especially during the period 1996–2015, although the situation was the opposite for the oldest group (85+ years). The contribution by old females aged 85 and above was more prominent than their male counterparts. It should be mentioned that the old males aged 85 and over had a negative influence on the overall change in male life expectancy in 1996–2005, and was much more obvious than that of females.

Apart from adults (aged 45–64) and the old population, the change in mortality in the 15–44 age group also exerted a positive impact on the growth in life expectancy in 1986–2015 in Hong Kong, even though the contribution declined over the two more recent decades. The contribution by infants was non-negligible as well, especially during 1986–1995, when a larger positive contribution occurred. In contrast with the age groups mentioned above, contributions by the 1–4 and 5–14 age groups were minimal, as they had very low mortality.

### 3.2. Contributions of Mortality Change by Causes of Death

Table 2 shows the contributions of mortality change to the improvement in life expectancy in Hong Kong by the leading causes of death from 1986 to 2015. Over the study period, for both males and females, substantial positive contributions to the increase in life expectancy in Hong Kong were observed in malignant neoplasms and circulatory diseases, including cerebrovascular diseases and heart diseases. Respiratory diseases also exerted an important influence on the total improvement in life expectancy, but the positive contributions of chronic lower respiratory diseases were partly offset by the negative contributions of pneumonia. Additionally, it was observed that a decline in mortality from external causes of death made a positive contribution to the health improvement in Hong Kong, which was more prominent in the case of males.

Compared with the diseases mentioned above, the contributions of other diseases—such as infectious diseases, digestive diseases and genitourinary diseases—were limited. For example, tuberculosis was no longer a leading cause of death, and thus it only made a small contribution to the increase in life expectancy in Hong Kong.

### 3.3. Contributions of Mortality Change by Age and Causes of Death

**Figure 2:**
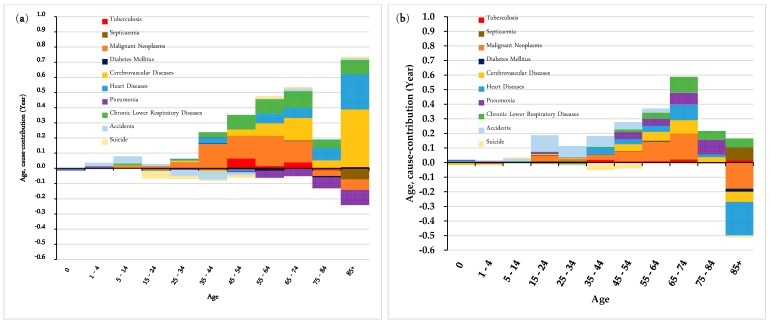

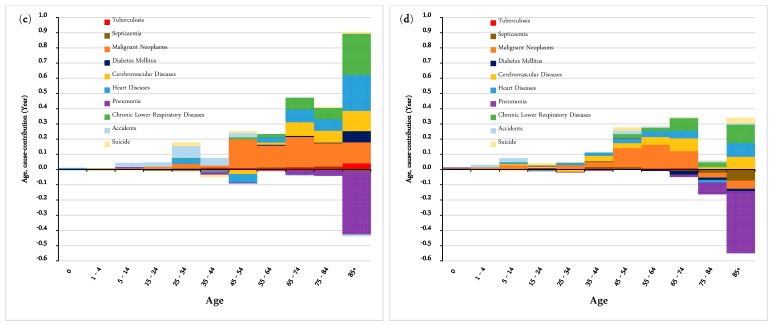

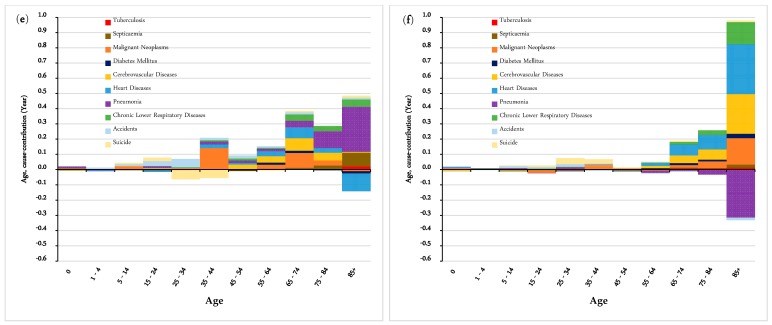
Arriaga’s decomposition of the change in life expectancy by age group and major causes of death in 1986–2015 in Hong Kong: (**a**) Contributions by males in 1986–1995; (**b**) Contributions by males in 1996–2005; (**c**) Contributions by males in 2006–2015; (**d**) Contributions by females in 1986–1995; (**e**) Contributions by females in 1996–2005; and (**f**) Contributions by females in 2006–2015.

Figure 2 presents the contributions of mortality change to the improvement in life expectancy in Hong Kong by age and causes of death during the period 1986–2015. In 1986–1995 and 1996–2005, for both males and females, more than 18 percent of the total increase in life expectancy in Hong Kong was due to a decline in mortality from malignant neoplasms, and this was mainly attributable to the mortality improvement in the adult and lower old age groups. During the period 2006–2015, however, the contribution pattern of malignant neoplasms changed, with males exerting a more prominent influence than females (50.1% = 0.887/1.77 in males, and 18.2% = 0.226/1.242 in females). In this period, both male adults (45–54 and 55–64 age groups) and older groups made remarkable positive contributions to the increase in male life expectancy, while the contribution of females was mostly from older age groups (especially those at advanced ages, who were responsible for 0.173 years out of the total increase of 1.242 years). By contrast, the mortality of malignant neoplasms rose in some younger female groups, offsetting its overall positive contributions.

The contribution patterns of circulatory diseases for both genders also changed over the past three decades, with their peaks shifting to older age groups, particularly in the case of old females at advanced ages. During the period 2006–2015, lower mortality from cerebrovascular diseases for females aged 85 years and above made obvious contributions to the total improvement in female life expectancy, more markedly than their male counterparts (7.5% in males and 20.9% in females), with a similar finding for heart diseases (13.4% in males, and 26.4% in females). However, it should be noted that the younger age groups exhibited an opposite trend, showing a deterioration in health among the younger population, even though this negative influence was weak.

On the contrary, although there was a positive rebound in the period 1996–2005, pneumonia contributed negatively to the total improvement in life expectancy. The main driver was the increase in mortality from pneumonia in the older population, especially among those at advanced ages. The situation was worse for both old males and females (aged 85 and over). To be more specific, during the period 2006–2015, females aged above 85 years contributed −25.3% (= −0.314/1.242) to the total change in female life expectancy, while males of the same age contributed −23.6% (= −0.417/1.77) to the overall growth in male life expectancy.

In terms of external causes of death, the change in mortality from accidents had a positive impact on the increase in life expectancy during the observed period. However, during 1996–2015, the contribution of accidents dropped from 0.364 years to 0.188 years in males, and from 0.179 years to 0.015 years in females. Positive contributions were primarily due to a decline in mortality in younger age groups, particularly among males.

## 4. Discussion

This study demonstrates an association between an improvement in life expectancy and a change in mortality by age and selected causes of death in Hong Kong. Our findings have certain practical implications; they can provide useful information for more effective allocation of health resources and more efficient implementation of health-related public policies in Hong Kong.

The results indicated that the improvement in health in both the adult and old age groups played a significant role in the increase in life expectancy for both males and females in Hong Kong over the period under study. However, in the latter years of the study period, it was observed that the contribution rate of the adult age groups dropped significantly, and the life expectancy advantage for both genders was then mainly driven by a decline in the mortality of the older population. Our findings concerning Hong Kong were consistent with existing studies in other countries such as Italy and Germany [4,7], where the primary contributions were also derived from the elderly.

The reason for the limited contribution of younger ages to the increase in life expectancy is related to the “rectangularization of the survival curve”. In the first half of the 20th century, mortality reduction in the younger age groups greatly increased life expectancy. Since the 1950s, however, a reduction in mortality among old people became the major contributor. The transformation of the survival curve strengthened the role of the elderly population in recent decades [15,16]. Besides, the “compensation law”—which has been mentioned in previous studies and states that a reduction in early mortality is compensated with a higher rate of change of mortality with age [17]—also explains why the increase in life expectancy had less magnitude than the mortality decline.

In addition, the contribution pattern varied across genders. In terms of age-specific contribution, the contributions of females at older ages were even more substantial than those of males, which was in line with the results of previous research [4,18]. On the other hand, the different contribution patterns for males and females resulted in different gains in their life expectancies over the distinct periods, as observed in Table 2.

The present findings showed that the decline in mortality from chronic diseases, such as malignant neoplasms, cerebrovascular diseases and heart diseases, made substantial positive contributions to the total improvement in life expectancy in Hong Kong between 1986 and 2015. A similar trend was observed in other countries, such as Italy and Germany, where the reduction in mortality from cardiovascular diseases and malignant neoplasms exerted important positive influence on their life expectancy gains [4,7]. In Japan, a rapid decline in mortality from cerebrovascular diseases also played a primary role in the improvement in Japanese life expectancy [18,19]. Therefore, the increase in life expectancy in Hong Kong over the past decades could partly be attributed to the reduction in mortality from those chronic diseases, especially among the older population.

Improvement in health in Hong Kong during the past decades is likely to be related to several factors. First, the well-established, accessible and affordable medical health system has played a crucial role in enhancing the health conditions of population in Hong Kong [20]. Besides, an increase in government expenditure in health care has promoted the development of medical and health services in Hong Kong [20]. Moreover, strategies which have focused on preventing chronic diseases may have further contributed to an effective control of chronic diseases. For instance, since 2008, the Hong Kong government has launched a strategic framework to prevent and control non-communicable diseases and has also formed a guiding committee to supervise its practical implementation [21], providing effective direction for prevention of chronic diseases in Hong Kong.

Furthermore, it should be pointed out that among older adults, especially among those at advanced ages, pneumonia was still noticeable in halting the increase in life expectancy in Hong Kong. Pneumonia can affect people of any age, but older people with a weak immune system tend to have a higher risk of developing the disease. According to Li et al. [22], during the period 2011–2015, old people aged 65 and over accounted for more than 75 percent of pneumonia-related hospitalizations in Hong Kong, which certainly caused a huge health and economic burden. With a rapidly accelerated ageing population in the coming decades [23], it is absolutely worth paying attention to the prevention of pneumonia in Hong Kong.

According to the present study, in the past few years, increasing prevalence of some chronic diseases among younger age groups has halted the improvement in life expectancy in Hong Kong, even though the influence was limited. Non-communicable diseases (NCDs) were principle health challenges around the world, and almost half of the deaths were among people younger than 70 years [24]. Compared with the elderly, those premature deaths that were primarily attributable to NCDs were within the most effective years of their work, thus causing substantial stress to both families and the national economy [25]. Adult mortality from NCDs and injury has also been discussed in previous research [26,27,28]. These studies mentioned the premature death from NCDs in Japan, the US and central and eastern European countries. Therefore, the health status of younger age groups should also be noted in Hong Kong.

This study has important implications for practical health interventions. The results reflect that secondary preventive strategies are still meaningful for the further reduction in mortality from chronic diseases in older age groups, as well as in younger age groups. It is generally accepted that dietary and behavioral factors, such as smoking and a lack of physical activity, are major factors for chronic diseases [29,30]; thus, a healthier lifestyle will play an important role in the prevention of chronic diseases. Precautionary measures are also crucial for the prevention of pneumonia or secondary bacterial infections. For example, the use of vaccination against influenza viruses was a fundamental means to reduce deaths from pneumonia caused by influenza infection [31]. On the other hand, since the elderly—especially those at advanced ages—have become healthier, with an increase in the number of elderly residents in Hong Kong in the future, medical treatment and health care needs will be big challenges to the health system.

Nevertheless, this study has some shortcomings. For instance, owing to the limitation of the available data, the last age group was 85 years and above. Since the health conditions of the older population (e.g., the 85–99 years) and even centenarians (the 100+ years) could be very different [10], it is necessary to further explore the health status of these additional specific age groups.

## 5. Conclusions and Future Work

This study reveals the growth mechanism of life expectancy in Hong Kong over the past decades. The results showed that, during the observed period from 1986 to 2015, the major contribution to the improvement in life expectancy in Hong Kong for both males and females was mainly attributable to the older population. The reduction in mortality from malignant neoplasms and circulatory diseases (e.g., cerebrovascular diseases and heart diseases) made substantial positive contributions to the increase in life expectancy, with the age groups making the greatest contributions shifting to the older ages in more recent years. However, these positive contributions were partly offset by a negative contribution of pneumonia, especially among those at advanced ages. Therefore, although Hong Kong has achieved a remarkable feat in improving the overall life expectancy, further measures can and should be taken to sustain its continuous improvement.

It is well known that the trend in life expectancy can be affected by multiple factors, such as socioeconomic state and availability of public health services [7]. Due to limitations in the data, we did not explore such issues here. In the future, this study could be further developed to explain the influence of such factors on the changing life expectancy.

## Figures and Tables

**Table 1:** Mortality rates for selected causes of death in 1986–2015 in Hong Kong (per 100,000).

Causes of Death	1986		1995		2005		2015	
	Males	Females	Males	Females	Males	Females	Males	Females
All causes	507.3	428.6	577.9	443.3	661.1	485.1	757.9	530.2
Infectious Diseases	17.2	10.8	20.3	15.4	18.1	13.8	20.1	14.9
Tuberculosis	11.1	3.8	10.9	3.4	6.3	1.8	3.9	0.7
Septicaemia	4.8	6.7	8.9	11.8	10.2	10.9	12.4	11.8
Other Infectious Diseases	1.3	0.3	0.6	0.2	1.7	1.1	3.8	2.4
Malignant Neoplasms	172.4	117.1	196.0	119.7	230.2	136.6	247.4	151.5
Endocrine Diseases	3.6	5.9	6.0	10.4	8.9	11.6	8.5	8.2
Diabetes Mellitus	3.3	5.4	5.5	9.7	7.5	10.0	7.0	6.4
Other Endocrine Diseases	0.2	0.5	0.5	0.7	1.4	1.5	1.5	1.8
Circulatory Diseases	134.8	136.4	143.9	140.3	157.4	145.0	159.2	119.0
Cerebrovascular Diseases	49.4	58.7	49.8	58.3	51.3	50.4	49.1	40.6
Heart Diseases	64.8	59.5	75.6	65.8	86.4	75.0	90.4	63.9
Other Circulatory Diseases	20.6	18.2	18.4	16.2	19.7	19.6	19.8	14.5
Respiratory Diseases	82.5	65.3	108.9	81.0	128.2	83.1	177.5	116.9
Pneumonia	28.9	28.3	55.7	52.7	70.3	57.2	124.9	96.3
Chronic Lower Respiratory Diseases	45.4	27.9	45.5	22.2	49.7	19.0	36.9	9.7
Other Respiratory Diseases	8.2	9.1	7.7	6.2	8.2	6.9	15.8	10.9
Digestive System	23.0	15.1	26.4	19.5	24.0	18.3	26.1	17.4
Genitourinary System	18.5	25.5	16.8	20.8	24.2	28.8	31.3	30.8
External Causes	34.9	21.5	37.2	17.1	38.4	19.1	35.3	17.3
Accidents	17.3	8.5	18.5	6.5	16.1	6.4	17.1	8.4
Suicide	13.5	10.4	16.1	9.7	18.9	10.9	17.4	8.6
Other External Causes	4.0	2.6	2.6	0.9	3.4	1.8	0.8	0.4

**Table 2:** Arriaga’s decomposition of the change in life expectancy by major causes of death in 1986–2015 in Hong Kong.

Causes of Death	Males			Females		
	1986–1995	1996–2005	2006–2015	1986–1995	1996–2005	2006–2015
All Causes	3.392	1.404	1.770	2.132	1.292	1.242
Infectious Diseases	0.041	0.150	0.078	−0.033	0.150	0.038
Tuberculosis	0.116	0.073	0.098	0.031	0.037	0.037
Septicaemia	−0.107	0.099	0.019	−0.074	0.129	0.031
Other Infectious Diseases	0.033	−0.022	−0.039	0.010	−0.016	−0.030
Malignant Neoplasms	0.613	0.279	0.887	0.391	0.312	0.226
Endocrine Diseases	−0.033	−0.020	0.071	−0.059	−0.009	0.066
Diabetes Mellitus	−0.018	−0.017	0.076	−0.058	0.003	0.053
Other Endocrine Diseases	−0.015	−0.003	−0.005	0.000	−0.012	0.013
Circulatory Diseases	1.452	0.202	0.832	0.675	0.192	1.069
Cerebrovascular Diseases	0.709	0.166	0.278	0.312	0.218	0.392
Heart Diseases	0.471	0.022	0.411	0.231	0.031	0.520
Other Circulatory Diseases	0.272	0.015	0.143	0.131	−0.056	0.157
Respiratory Diseases	0.455	0.631	−0.166	−0.037	0.695	−0.225
Pneumonia	−0.277	0.302	−0.528	−0.502	0.519	−0.351
Chronic Lower Respiratory Diseases	0.498	0.300	0.458	0.291	0.159	0.197
Other Respiratory Diseases	0.235	0.029	−0.096	0.174	0.017	−0.071
Digestive System	0.092	0.217	0.117	−0.039	0.083	0.124
Genitourinary System	0.362	−0.032	0.075	0.314	−0.066	0.121
External Causes	0.017	0.269	0.229	0.241	0.135	0.146
Accidents	0.012	0.364	0.188	0.093	0.179	0.015
Suicide	−0.067	−0.125	0.028	0.078	−0.073	0.108
Other External Causes	0.072	0.030	0.013	0.070	0.029	0.022

## Appendix B

**Table A1:** Codes for the selected leading causes of death in the different International Classification of Diseases (ICD) revisions.

Causes of Death	ICD 9	ICD 10	ICD Update
	(1986–2000)	(2001–2009)	(2010 Onwards)
Infectious Diseases	001–139	A00–B99	A00–B99
Tuberculosis	010–018, 137	A15–A19, B90	A15–A19, B90
Septicemia	38	A40–A41	A40–A41
Malignant neoplasms	140–208	C00–C97	C00–C97
Endocrine Diseases	240–278	E00–E89	E00–E89
Diabetes Mellitus	250	E10–E14	E10–E14
Circulatory Diseases	390–459	I00–I97, I99	I00–I97, I99
Cerebrovascular diseases	430–438	I60–I69	I60–I69
Heart Diseases			
Respiratory Diseases	460–519	J00–J98	J00–J98
Pneumonia	480–486	J12–J18	J12–J18
Chronic Lower Respiratory Diseases	490–494,496	J40–J47	J40–J47
Digestive Diseases	520–579	K00–K92	K00–K92
Genitourinary Diseases	580–629	N00–N99	N00–N99
External Causes	E800–E999	V01–Y89	V01–Y89
Accidents	E800–E949	V01–X59	V01–X59
		Y40–Y86	Y40–Y86
		Y88–Y89	Y88–Y89
Suicide	E950–E959	X60–X84	X60–X84
